# Noises in the Ears

**Published:** 1890-12-27

**Authors:** 


### NOISES IN THE EARS.

Dr. Macnaughton Jones's paper on "The Etiology of *Tinnitus Aurium*," which caused considerable discussion at the last meeting of the British Medical Association, has recently been published. Dr. Macnaughton Jones has recorded the following noises in the ears as complained of by his patients: Buzzing, sea-roaring, sounds as if trees were agitated by the wind, singing of kettle, bellows, bee murmur, noise of a shell bursting, puffing, thumping, continued beating, crackling sounds, whistle, furnace blowing, rain falling, distant thunder, chirping of birds, waterfall, mill-wheel, music, and bells. But Dr. Laurence Turnbull says we may reduce all noises in the ears to three classes: (1.) Constant rushing, knocking, or pulsating noises. (2.) What are termed moist sounds, as gurgling, bubbling, boiling, singing, and whistling. (3.) Dry roaring and ringing noises. It appears that tinnitus aurum may exist without any impairment of hearing, or it may be connected with some disease of the ear, acute or chronic, which causes impairment of hearing. According to Dr. Jones, the recognized causes of tinnitus, when the hearing is normal, are recent sea-bathing, fever, alcoholic excess, topical causes, cardiac weakness, nasal obstruction, puerperal septicæmia, irregular catamenia, mental strain, over-study, menorrhagia, Bright's disease, the menopause, albuminuria, use of quinine, retroflexion of the uterus with neurosis, pharyngeal catarrh, neurotic temperament, and tobacco. And Mr. Laurence Turnbull says there are certain habits of body which induce the noises. These are anything which congests the parts, as the free use of stimulants or tobacco, great bodily and mental fatigue, reading with a gas burner or an oil lamp near the head. There are also mental influences, both external and internal, as worry, and a hot, close room. Sometimes tinnitus is associated with vertigo. Dr. Jones observes that "certain disturbances in the equilibration of the labyrinthic fluid, or reflex irritations of any part of the nervous apparatus of the labyrinth, or irritation of the auditory nerve nuclei, may cause vertigo to accompany tinnitus." For many years our knowledge of tinnitus aurum was very obscure, and noises in the head or ears were classed as nervous. This is now recognized not to be the case. As before observed, tinnitus is frequently connected with well marked ear disease. And Dr. Turnbull states that in the *post mortem* examinations of persons who suffered from tinnitus, without any known disease of the ear, on dissections of the petrous bone there have been found diseases of the semicircular canals, ecchymoses in the vestibule, hyperæmia of the cochlea, or general enlargement and fulness of vessels of the labyrinth. There are, however, cases of purely nervous tinnitus. We have known tinnitus to occur in several forms, *instead* of an attack of ague. That is, ague has occurred one day, and the next day at the same hour tinnitus has occurred. We have also known tinnitus to occur instead of ague every day at about the same hour, and to last about the time of an ordinary attack of ague. Yet these patients recovered perfectly without any defect of hearing.

